# Risk factors for not completing health interventions and the potential impact on health inequalities between educational groups – a mixed method study from Denmark

**DOI:** 10.1186/s12939-016-0344-6

**Published:** 2016-03-31

**Authors:** Nanna Kure-Biegel, Christina Warrer Schnohr, Anette Lykke Hindhede, Finn Diderichsen

**Affiliations:** Section of Social Medicine, Institute of Public Health, University of Copenhagen, Gothersgade 160, Copenhagen, DK-1014 K Denmark; Department of Learning and Philosophy, Aalborg University, A C Meyers Vaenge 15, DK-2450 Copenhagen, SV Denmark; Health Promotion Research, Steno Diabetes Center, Niels Steensens Vej 2, Gentofte, DK-2820 Denmark

**Keywords:** Health inequity, Tertiary prevention, Lifestyle factors, Interventions, Health policy

## Abstract

**Background:**

Individual-based interventions aim to improve patient self-management of chronic disease and to improve lifestyle among people at high risk, to reduce the prevalence of diseases contributing to health inequality. The present study investigates risk factors for uncompleted health interventions, via a combination of quantitative and qualitative methods.

**Methods:**

From a health centre in Copenhagen, questionnaire data on educational level, gender, age, and cohabitation status from 104 participants in health interventions were used to examine risks for dropout. Qualitative telephone interviews further investigated risk factors among 17 participants who were registered as uncompleted.

**Results:**

Our findings show that there is a significantly higher prevalence of uncompleted courses among participants below age 60 (OR 3.38, 95 % CI 1.08; 10.55) and an insignificantly higher prevalence among people with low education (OR 1.82, 95 % CI 0.66; 5.03). Qualitative elaboration of these findings points to low self-control in jobs and a higher degree of comorbidity and treatment of diseases among the lower educated as determinants for not completing, but not lower motivation or less positive attitude toward the intervention itself.

**Conclusions:**

This study indicates a social difference in dropout, and if dropout is to be prevented, there is a need to acknowledge factors such as organization of the intervention, lack of job flexibility, and comorbidity. If these factors are not addressed, people with low socioeconomic status will most likely have reduced opportunities for making healthy choices, in this case, completing the intervention, and this may increase health inequality.

## Background

Fair distribution of health and well-being are important social goals in many countries [1]. Fair distribution implies health equity, which is defined as *the absence of avoidable or remediable differences among populations or groups defined socially, economically, demographically or socially* (1: 7).

In Denmark, there has been increasing focus on health inequality, both at the national and local levels. Despite the fact that economic inequality is relatively small in Denmark compared to other countries, there are large socioeconomic differences in mortality, differences in the prevalence of disease, inequities in access to health care, and differences in quality of life, depending on education, income, area of residence, and gender [2].

Due to a revision in health legislation effective 1 January 2007, the 98 Danish municipalities assumed the main responsibility for preventive services and health interventions to promote a healthy lifestyle, in collaboration with Denmark’s regions and general practitioners (GP’s) [3].

More than two-thirds of Denmark’s 98 municipalities have chosen to establish health centres, which offer a variety of interventions aimed at individual groups [4]. In the municipality of Copenhagen, health centres have placed great emphasis on health inequalities, since these are quite large across the city. In the centre of the city, life expectancy is 80.2 years, whereas life expectancy in Noerrebro is only 73.3 years. In certain areas of Copenhagen, average life expectancy is 69.4 years for men, which is the same as the average life expectancy for men in Denmark 50 years ago [5]. The great and increasing inequality in health has led to strengthened efforts in policy and practice with the goal of reducing this inequality [6].

Interventions are often planned with a focus on particular risk factors, such as unhealthy diet, smoking, excessive alcohol consumption, and sedentary lifestyle [7]. The strong focus on these risk behaviours is based on firm evidence that inequality in the prevalence of these risk factors forms a large part of the relatively low life expectancy in lower socioeconomic groups and contributes substantially to social inequality in health [8].

From other health promotion interventions, it is observed that dropout rates are high in lifestyle programmes, and little is known about predictors for compliance and adherence [9]. Some studies show that there is an inverse correlation between attendance and risk for disease [10], and others indicate that the reasons for dropout are presence of disease or participation in other medical treatments [11]. If this is true, health promotion interventions will increase, rather than decrease, the inequity in health. But since few interventions targeting lifestyle factors have been evaluated [12], it remains unknown what effect the interventions have, and hence whether Danish municipalities are creating a setting for a healthy lifestyle and reducing health inequality. The present study examines the hypothesis that there is a social difference among participants not completing interventions targeting lifestyle factors, and it aims to elucidate the barriers to non-completion. Social status difference, between those who completed and those who did not, is measured via data on education and cohabitation status. We used interviews examining the participants’ views on individual, interpersonal, and organizational factors for not completing the intervention. We also discuss whether lifestyle interventions potentially contribute to the reduction in inequality in health.

## Methods

### Study area, population, and intervention

The catchment area is Vanloese/Broenshoej/Husum (denoted VBH), one of 10 districts of Copenhagen, with approximately 60,000 inhabitants above 16 years of age. VBH has a relatively high proportion of citizens with low education or low income, a high level of unemployment, and a high proportion of small housing. The health promotion centre in VBH offers a range of health-promoting interventions to reduce smoking, improve dietary behaviour and to promote weight loss. All participation is voluntary for citizens of VBH, and participants can be referred to an intervention by their doctor, by a hospital, or they can obtain service via walk-in. The intervention is meant to last 6 to 12 weeks, with activities once or twice per week. At the start and end of each intervention, the participant has a scheduled one-hour meeting with a health professional to analyse the needs of the participant to pursue a healthy lifestyle. In collaboration with the health professional, the participant can choose to attend one or more activities, such as personal or group sessions, workouts, non-smoking sessions, or hands-on activities such as cooking. An important part of the intervention is that the health professionals help participants to continue the changed behaviour post-intervention.

Recruitment to the present study is from a sub-study from a research collaboration between VBH and Steno Diabetes Center Prevention Research, with the purpose of examining the socioeconomic position of citizens attending the VBH centre [13]. Collection of data occurred between December 17, 2012 and September 31, 2013 where 376 citizens were asked through a questionnaire about age, gender, education, and marital status. 198 (52 %) citizens responded to the questionnaire, whereas 180 ticked a box with the option to be contacted for related research projects in the future. Notes from health personnel at the centre gave information about cancellation of first meeting. Cancellation of first meeting includes that participants did or did not show up at the health centre at the appointed time or called in to reschedule for a new first meeting. These 180 participants had initially been categorized by health personnel into three categories based on the rationale for applying or being referred to the health intervention and to obtain the most optimal combination of like-minded participants in each course of intervention. Twenty-five participants were not ill, but sought help to improve their overall health status, for example, to stop smoking. These participants were classified as primary prevention. Forty-six participants were attending due to early detected risk factors that they aimed to reduce, for example, weight loss. These participants were classified as secondary prevention. 104 participants had diabetes, heart disease, or chronic obstructive lung disease (COLD) and were classified as tertiary prevention. There was no information on the rationale for applying from five participants.

**Fig. 1:**
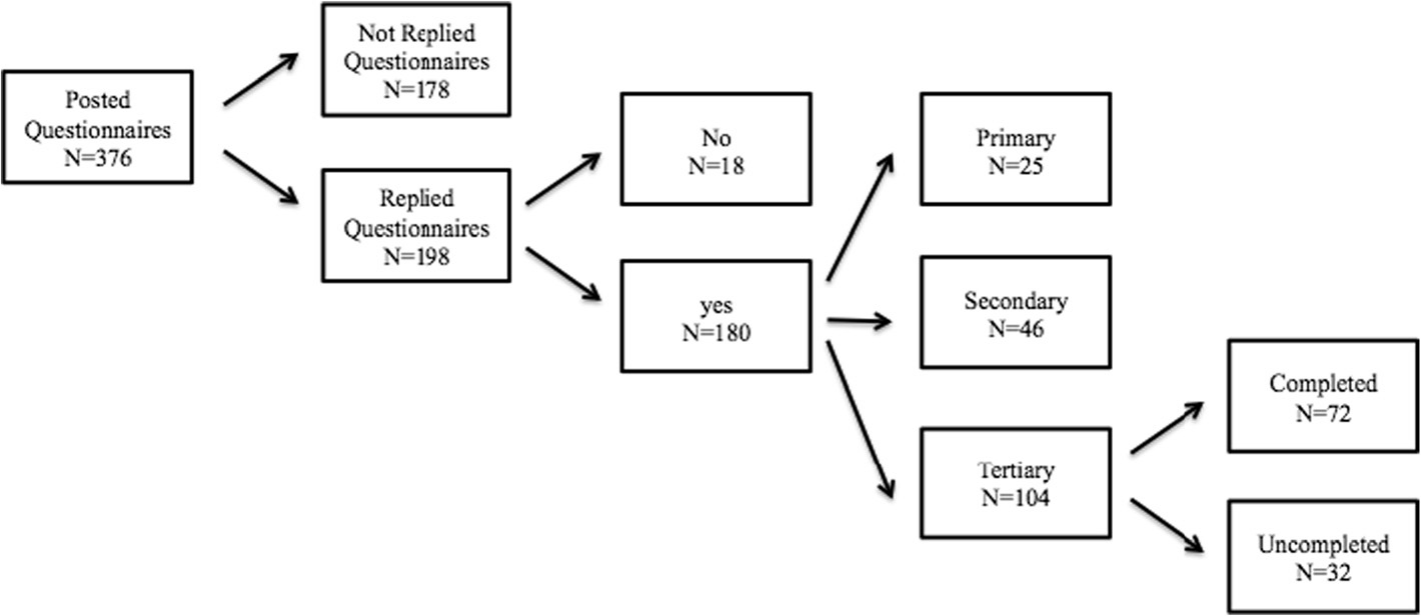
Recruitment of respondents

The age distribution for the primary group was 20 to 76 years, for the secondary group, it was 24 to 94 years, and 39 to 86 years for the tertiary group. Due to heterogeneity of the primary and secondary groups, the participants from the tertiary group, who all had chronic disease, were included (*N* = 104). Figure 1 illustrates recruitment of participants. Of the 104 participants, 72 (69 %) completed their courses and 32 (31 %) did not.

### Outcome variable: uncompleted interventions

The outcome in question is whether participants had completed their health intervention. Two health professionals assessed whether each participant completed the planned intervention on the basis of notes from health workers. Uncompleted could mean the participant informed the centre of a decision to stop participating in the course, the participant was absent several times, or the health worker was hindered from getting in contact with the participant via the phone number provided in the participant’s journal file.

### Statistical analysis

Data were analysed using SPSS version 22. The quantitative analyses are a comparison of completion with respect to inequality between population groups based on education, gender, age, cohabitation status and first interview cancellation, conducted via a Chi^2^ test and a multivariate analysis examining models of logistic regression analyses. Four different models were calculated, using a stepwise method where controlling for different covariates per model.

### Recruitment for interviews

The 32 participants who did not complete their intervention were contacted by telephone in March 2014 to address the barriers they experienced in completing the course. Out of these 32 respondents, 1 had died, 2 claimed to be too sick, 2 had changed phone number and could not be reached, 2 did not speak Danish sufficiently, 6 could not be contacted, and 2 refused, leaving 17 completed interviews.

### Interviews

The qualitative data are based on telephone interviews with a focus on examining barriers as well as promoting or inhibiting factors for completion. The interviews were conducted on the basis of an interview guide, with open questions related to elucidating factors on individual, interpersonal, and organizational elements influencing participation in and completion of the intervention. The questions covered themes on the individual level about the participants’ attitude toward and knowledge about the health centre; on the interpersonal level, questions were about experiences from being part of the intervention, interacting with the health professionals, and how family and friends reacted to participation; on the organizational level, questions were about how the participants’ everyday life matched up with being part of the intervention. All interviews were done by telephone after receiving informed consent from the participant. The interviews lasted approximately 15 min each and were recorded and transcribed. Transcripts were read and subthemes identified and agreed upon under three main themes: individual, interpersonal, and structural factors promoting or inhibiting participation. Repeated reading of the transcripts and field notes led to identification of the most prevalent barriers for completion.

## Results

Table 1 presents the demographic characteristics of the 104 participants from the tertiary prevention group and the distribution of the characteristics in relation to completed or not completed intervention.

**Table 1:** Summary of characteristics of participants with a chronic disease

	Categories	N	Completed		
			Yes (%)	No (%)	*p*-value
Education (*N* = 97)	Low	55	61.8	38.2	0.13
	High	42	76.2	23.2	
Gender (*N* = 104)	Woman	51	64.7	35.3	0.32
	Man	53	73.6	26.4	
Age (*N* = 102)	<60	22	50	50	0.03
	>60	80	73.8	26.3	
Cohabitation status (*N* = 100)	Living together	54	61.1	38.9	0.06
	Living alone	46	78.3	21.7	
Cancellation of first meeting (*N* = 104)	Cancelled	16	43.8	56.3	0.01
	Not Cancelled	88	73.9	26.1	

Low education: no basic schooling, still at school, college, commercial college

High education: short-, medium-length education, or more than 4 years of education

First meeting: Participants not showing up, cancelling or rescheduling first meeting at health centre

The quantitative data show that there is a non-significant tendency toward a higher prevalence of uncompleted courses among the lower educated, among women, and among participants living together. Further, there is a significantly higher prevalence of uncompleted courses among participants under age 60, as well as for participants who cancelled their first scheduled interview.

Table 2 shows the results of the four logistic regression analysis: Model 1 is the univariate analysis of education on completion. The following three models are adjusting for the following confounders: gender and age (Model 2), cohabitation status (Model 3) and first interview cancellation (Model 4). All four models shows and insignificant lower OR for people with low education for completing compared to people with high education (Table 2). The fully adjusted model (Model 4) shows an insignificant increased OR of 1.82 for completing for people with high education, and an increased odds ratio (OR) for men of 2.14 (95 % CI 0.76; 6.03), and for participants 60 years and older the increase is 3.38 (95 % CI 1.08; 10.55). Participants who showed up from the first meeting had an OR of 2.77 (95 % CI 0.80; 9.57) for completing. Compared to Table 1, only variable age is significant.

**Table 2:** Analytical quantitative analyses of completion of health promotion program with respect to inequality between population groups

	Groups	Model 1	Model 2	Model 3	Model 4
		OR (95 % CI)	OR (95 % CI)	OR (95 % CI)	OR (95 % CI)
Education	High/low	1.96 (0.81;4.84)	2.22 (0.86;5.73)	1.86 (0.70;4.93)	1.82 (0.66;5.03)
Gender	Man/woman		1.75 (0.70;4.34)	2.12 (0.79;5.66)	2.14 (0.76;6.03)
Age	≥60/< 60		3.49 (1.23;9.88)	3.02 (.03;8.86)	3.38 (1.08;10.55)
Cohabitation status	Living alone/living together			2.17 (0.79;5.98)	2.39 (0.83;6.89)
Cancellation of first meeting	Not Cancelled/cancelled				2.77 (0.80;9.57)

Low education: no basic schooling, still at school, college, commercial college

High education: short-, medium-length education, or more than 4 years of education

Cancellation of first meeting: Participants not showing up, cancelling or rescheduling first meeting at health centre

### Characteristics of participants not completing the intervention

Table 3 describes demographic characteristics for the 17 interviewees not completing the intervention compared to the 15 participants not interviewed. Through chi^2 test it was tested whether the 17 interviewed were representative regarding the chosen parameters for the 15 people not interviewed who neither completed the intervention.

**Table 3:** Descriptive analyses of 17 persons interviewed and 15 not interviewed

	Categories	N	Interviewed		
			Yes (%)	No (%)	*p*-value
Education (*N* = 32)	Low	23	52.2	47.8	0.59
	High	9	55.6	44.4	
Gender (*N* = 32)	Woman	18	50.0	50.0	0.11
	Man	14	57.1	42.9	
Age (*N* = 32)	<60	11	72.7	27.3	0.01
	>60	21	42.9	57.1	
Cohabitation status (*N* = 32)	Living together	22	59.1	40.9	0.27
	Living alone	10	40.0	60.0	
Cancellation of first meeting (*N* = 32)	Cancelled	9	33.3	66.7	0.16
	Not Cancelled	23	60.9	39.1	

Low education: no basic schooling, still at school, college, commercial college

High education: short-, medium-length education, or more than 4 years of education

Cancellation of first meeting: Participants not showing up, cancelling or rescheduling first meeting at health centre

A statistical difference is seen among the interviewed and not interviewed with respect to age, but there is no statistical difference with respect to the rest of the parameters between the interviewed and the not interviewed.

#### Promoting and inhibiting factors

The qualitative data first and foremost show that approximately half the participants (7 out of 17) did not consider their health intervention as ‘uncompleted’. All participants came up with several factors, each either impeding or advancing participation, and consistent patterns are evident in the form of repeated answers. Often, participants had a personal understanding of one or several factors as being decisive when responding to barriers to completing a course. The qualitative interviews show a set of themes as either promoting or inhibiting factors at an individual, interpersonal or organizational level; these themes are illustrated in Table 4.

**Table 4:** Qualitative analysis of promoting and inhibiting factors for completing health intervention

Themes	Promoting or inhibiting
Individual	
• Desire for healthier lifestyle	• Promoting
• Positive attitude towards health centre	• Promoting
• Attendance at health centre gives increased knowledge about healthy lifestyle	• Promoting
• Self-determination among men	• Promoting
Interpersonal	
• Experiencing positive responses from health professionals	• Promoting
• Social support from others	• Promoting
• Changed lifestyle due to health intervention	• Promoting
• Age difference among participants	• Inhibiting
Organizational	
• Lack of job flexibility	• Inhibiting
• Opening hours of health centre	• Inhibiting
• Lack of coordination with visits to hospitals	• Inhibiting
• Taking care of sick family members	• Inhibiting

#### Individual factors

When asked about individual factors, participants emphasized that self-determination propelled them to complete their health interventions. Several participants, 10 out of 17, expressed that a desire for a healthier lifestyle was of great value, and they perceived the centre as a promoting factor to achieve this via increased knowledge of health-related issues and strong support, as illustrated by the following quotes:

*‘We had tons of good advice about healthy dieting, what you should not eat and stuff like that. It was very fruitful (..) also when we were in the gym’* (non-completing participant).

*‘The importance of living healthy has been made so very clear to me, I mean really, I have actually become aware of how crucial it is’* (non-completing participant).

In particular, the men expressed a great deal of self-determination in completing their health intervention; once they had been told that completion would benefit them, they were determined to do so and had a positive attitude toward the intervention. Other promoting factors include specialized knowledge gained from physiotherapists, nurses, and occupational therapists, among other practitioners, employed at the centre. 13 participants expressed a positive attitude toward the centre and underscored high expectations as to course content and the personal benefit gained.

#### Interpersonal factors

Fifteen participants expressed positive experiences from interaction, social support, and response from the health professionals and the other course participants. An example of this is illustrated by the following quote:

‘*It [intervention] is a really good idea, they [the health professional] are helping us in a manner that fits our specific need’* (non-completing participant).

Twelve participants expressed that they experienced a positive response to the intervention by way of a changed lifestyle and that this change was maintained after the end of the course, either at home or in local facilities. Of the interviewed, 8 were under 60 years old and 3 of these pointed out that most courses had older participants, which was demotivating and an inhibiting factor for the younger age groups.

#### Organizational factors

For participants still working, 9 participants reported that it was a significant barrier to participate in a course during working hours, as they reported no job flexibility to take time off. Another barrier was the hours of the health centres: the latest appointment was around 3 pm, which was problematic for people who work. The chronically ill had many visits to doctors or treatments at a hospital; the treatments did not have any joint coordination with the intervention at the health centre. Nine participants were highlighting this theme and it is exemplified through the following quote:

*‘It was every day, it started to tip over with letters from hospitals and something like that, I go to three hospitals, so they all have different appointments that need to be respected’* (non-completing participant).

Several interviewees had other diseases and four participants drew attention to the fact that they were also taking care of sick or older members of the family, and had to plan their needs as well as their own, prioritizing the family. The fact that there was no coherence between treatments was a stressful and therefore inhibiting factor.

## Discussion

Our study set out to explore the hypothesis of a social difference in dropout in a health intervention and to elucidate reasons for people dropping out. Our main findings are that dropout is related to young age and surrounding factors, such as low job flexibility and lack of coordination with other treatments. Further, low education, female sex, cohabitation and cancellation of first interview are associated with dropping out. Due to lack of statistical power, the results, except for that of age, are not statistically significant.

The significantly higher prevalence of uncompleted courses among participants aged below 60 is also seen in other studies [11, 14]. Dropout associated with low education is in line with other studies showing that non-participation rates are higher for people with low socioeconomic status [15, 16]. Participants pointed out factors determining dropout, especially organizational factors, such as low personal job flexibility, opening hours of the centre, and lack of coordination with other treatments. People with lower socioeconomic status more often have low levels of control in their jobs [17], and thus lower job flexibility.

Non-completers were characterized by high motivation, a positive attitude towards the centre and interpersonal factors, such as rewarding interaction with health professionals. These characteristics were, however, not sufficient to complete the intervention. Earlier studies also point to these factors as determinants for participating in health interventions, but not necessarily as determinants for completion [9].

### Methodological considerations

One limitation of this study is a possible misclassification in the outcome, since some participants who were categorized as having an uncompleted intervention themselves thought the course was completed. The categorization was done by a health professional, who spent approximately one hour per participant going through all available files, recording the most valid outcome and resulting in the least possible misclassification bias.

Another limitation to this study is that ‘completed interventions’ is used as an indicator for changed behaviour, which is the aim of the study. But not completing the intervention does not need to be associated with lack of changed health behaviour. A qualitative study by Jones [18] shows that patients not participating in the studied health intervention adhered to another intervention or dropped out of the intervention, since health behaviour already had been changed.

A third limitation to the study is the low number of participants in the qualitative interviews; only 53 % of the participants not completing the intervention were interviewed, even though statistical analysis (Table 3) was made to ensure no selection bias among the interviewed, it is hard to conclusion due to lack of power in the analysis.

### Implications for future health interventions

The present study includes interpersonal and organizational factors, which provide a supplementary perspective to the knowledge on understanding participation and eventually reducing health inequality; that is, more distal factors also play a role as barriers to completion of health interventions. Future research on how to incorporate this knowledge into practise should be of high priority for future health interventions and for the goal of reducing inequality in health.

Whether the same social difference is seen in primary and secondary prevention is a topic for future research, but campaigns against smoking and campaigns for physical activity and improved diet are adopted primarily by people with high socioeconomic status; and dropout rates are higher for people with low socioeconomic status. However, this area is characterised by inconsistent scientific evidence on the effect of interventions directed toward specific groups [19], which points to the challenge of the most optimal approach and policy in this area.

Health centres can meet this need by, for example, offering interventions during later hours than that in the VBH centre, 4:30 pm. In this way, the centre may be able to take the social difference in job control into account. The need for coordination with other treatments is more difficult for the health centre to meet, since it is not in contact with hospitals, general practitioners and other health centres, to coordinate treatments or prevention courses for specific participants, and since people with low socioeconomic status (SES) often have more diseases and there is heightened participation in treatment and preventive health interventions [20]. For this group, it is more invasive, mentally overwhelming and more time consuming in everyday life than for the group of people having only one disease. The fact that several treatment and health interventions may be taking place simultaneously is important to take into account when planning health interventions to heighten participants’ ability to participate in and complete health interventions.

## Conclusion

The conclusion of this study is that intervention aimed at the individual is successful in providing participants with tools and knowledge about their disease and how to handle it, which they report on very positively in the interviews. If dropout is to be prevented, there is a need to acknowledge factors such as organization of the intervention, lack of job flexibility and comorbidity, which all have a social difference. If these factors are not addressed, people with low socioeconomic status will most likely have reduced opportunities to make healthy choices – in this case, completing the intervention. When this happens, it is possible that health interventions may result in the ‘inverse prevention’ or ‘inverse care law’ stated by Tudor Hart [19], which favours those who have the best health.
